# Advanced BCl_3_-Driven Deep Ion Etching of β-Ga_2_O_3_ for Precision High-Aspect-Ratio Nanostructures

**DOI:** 10.3390/s25216609

**Published:** 2025-10-27

**Authors:** Badriyah Alhalaili

**Affiliations:** 1 Nanotechnology and Advanced Materials Program, Energy and Building Research Center, Kuwait Institute for Scientific Research, Safat 13109, Kuwait; bhalaili@kisr.edu.kw; 2 American Romanian Academy of Arts and Sciences, Citrus Heights, CA 95616, USA

**Keywords:** dry etching, Ga_2_O_3_, etching rate, BCl_3_

## Abstract

Gallium oxide-based devices are critical in various applications, including industrial safety, the gas and petroleum sectors, and research environments. However, the deep etching process has not been thoroughly explored. Key parameters such as etching rate, selectivity, uniformity, isotropic/anisotropic behavior, and surface properties all influence the effectiveness of the etching process and its reproducibility. This research was motivated by the need for efficient fabrication processes, particularly in applications where sensors must operate in harsh environments, due to their instead of owning to low leakage current density of their power devices. In this study, we studied a deep etching technique for Ga_2_O_3_, focusing on the chemical stability of the two planes and identifying suitable protocols that could enhance etching depth via a dry-etching process. A deep ion-etching process for Ga_2_O_3_ was successfully developed, achieving deep etches of 6.97 µm in the Ga_2_O_3_. These advancements pave the way for high-aspect-ratio Ga_2_O_3_ nanostructures, offering new possibilities for robust nanosensors in harsh environments.

## 1. Introduction

Gallium oxide (Ga_2_O_3_) has percolated through many recent technologies and emerged as a pivotal semiconductor in modern technological advancements, garnering significant attention across various scientific disciplines due to its exceptional properties [1,2]. With an ultra-wide bandgap of approximately 4.8 eV, a high-breakdown electric field exceeding 8 MV/cm, and an electron mobility of approximately 150 cm^2^/V·s, Ga_2_O_3_ presents a compelling alternative for a range of applications, particularly in semiconductor research. Ga_2_O_3_’s cost-effectiveness and ease of manufacturing position it as a promising substitute for traditional materials such as silicon carbide (SiC) and gallium nitride (GaN) [3,4,5,6]. Ga_2_O_3_-based materials are some of the best ultra-wideband gap materials (UWBGs) and show great potential for safe and long-term sensing in harsh environments. Recently, Xinyi et al. showed the first thermal neutron detector based on a large-area (9 mm^2^) p-NiO/β-Ga_2_O_3_ heterojunction diode [7]. Additionally, the development of 3D-fabricated devices may lead to the efficient detection of alpha particles by their energy. The fabrication of 3D nanostructures using Ga_2_O_3_ is attracting attention for increasing the performance of devices, notably, in radiation, high-power electronics, and photonics applications, due to its high surface-to-volume ratio [1,6,8].

The etching processes utilized in the fabrication of Ga_2_O_3_ devices are critical for optimizing device performance. Wet-etching techniques can etch along specific crystallographic directions, such as in (100), (010), and ($\bar{2}$01) [9,10,11,12], which leads to etching in a slightly vertical direction [12]. However, these techniques are limited by their inability to achieve high aspect ratios in micro- or nanostructures, which adversely affects the uniformity and reproducibility required for advanced electronic and photonic devices [1]. These nanostructures facilitate photoenergy harvesting in optoelectronic sensors, e.g., UV detection for safety in petroleum sectors [1,2,3,4,5], by amplifying photo-generated carriers with an increased junction area. Consequently, there is growing interest in developing alternative dry-etching processes for single-crystal Ga_2_O_3_ to assess chemical stability and identify appropriate etchants that can enhance etch rates effectively.

While wet etching offers advantages such as minimal surface damage, high selectivity, and throughput, it is constrained by several factors that include (i) uncontrollable isotropic etching, which makes it difficult to introduce micro- and nanostructures, and (ii) the disposal of significant quantities of toxic chemical solvents. In contrast, dry-etching techniques, particularly deep reactive-ion (DRI) etching, facilitate anisotropic etching, providing enhanced control and reproducibility. DRI etching is currently used to fabricate anisotropic nano- and microstructures and involves material removal facilitated by neutral gas atoms in a vacuum chamber, although research into the optimal gas chemistries and etching mechanisms specific to Ga_2_O_3_ remains ongoing [1].

Despite the growing research and body of literature, a comprehensive understanding of Ga_2_O_3_ etching processes remains elusive, with numerous challenges associated with adjusting etching parameters [10,11,13,14]. Key variables influencing etching outcomes include the etch rate, selectivity, uniformity, directionality (isotropic versus anisotropic), surface quality, and reproducibility. Thus, researchers are actively seeking to establish optimal etching procedures that ensure chemical stability and enhance etch rates for Ga_2_O_3_.

In nanofabrication, dry etching involves gases or plasmas. It is a highly anisotropic, controlled process of the etching direction, with high uniformity [15,16,17]. Accordingly, dry etching is an important step for forming patterns on the surface of Ga_2_O_3_-based materials. Dry etching is the process of implementing plasma or ion beams to fabricate 3D nanostructures on the surface of a semiconductor, which are highly desirable for optimizing the nanosensors’ detection mechanism. The fabrication of 3D nanostructures is greatly beneficial for increasing the charge detection rate, leading to faster response and increasing the devices’ sensitivity. They are considered very effective and ideal for high-performance sensing applications [18].

However, one of the fabrication challenges encountered when dry etching Ga_2_O_3_-based materials is that they can have poor etch selectivity, which affects the difference between the etching rate of the Ga_2_O_3_ surface compared with the outside mask. This may cause the mask to be etched away before the structure is fully developed, thus ruining the entire process [19]. In addition, some unwanted defects can form on the surface from the ion bombardment, causing a break in the bonds. This leads to surface roughness and below-surface defects from the etching process [20].

Vertical devices such as β-Ga_2_O_3_ trench metal-oxide semiconductor (MOS) diodes and fin field-effect transistors employ deep-etching techniques to mitigate the intense electric fields in surface areas. However, the process of deep etching can lead to plasma damage due to inductively coupled plasma (ICP) reactive-ion etching (RIE). Publications dedicated exclusively to the fabrication of high-aspect-ratio nanostructures using wide-bandgap materials such as Ga_2_O_3_ are lacking. These structures are particularly valuable in the development of nanosensors, promising improved sensing mechanisms through increased profile depth, which is anticipated to enhance performance in extreme environmental conditions.

In this study, we present the development of a deep ion-etching process for β-Ga_2_O_3_, using BCl_3_-based inductively coupled plasma (ICP) reactive-ion etching (RIE). Unlike [21,22], which discuss mixtures for moderate depths, our pure BCl_3_ ICP-RIE with Ar-interrupted cycles and SUEX overcomes small-substrate lithography barriers, achieving unprecedented 6.97 µm depths for vertical devices. Several device applications have been obtained using BCl_3_-driven deep ion etching of β-Ga_2_O_3_, such as field-effect transistors [23] and Schottky barrier diodes [24]. Unlike prior work on etching or wet-etching techniques [10,11,13,14,25], this research systematically investigated the chemical stability of Ga_2_O_3_ (010) and ($\bar{2}01$) planes, optimizing etching parameters to enhance etch depth, selectivity, and surface quality.

## 2. Materials and Methods

### 2.1. Surface Fabrication by Photolithography

In this study, 5.00 mm × 5.00 mm Sn-doped Ga_2_O_3_ substrates, sourced from Tamura Corporation in Tokyo, Japan, were employed for the anisotropic etching procedure. Sn-doped substrates with distinct orientations, specifically cleaved Ga_2_O_3_ (010) and Ga_2_O_3_ ($\bar{2}01$), were used. Ga_2_O_3_ Sn doping was achieved at 0.03%. A photolithography mask was designed and fabricated by the vendor. The experiments were conducted using advanced fabrication and characterization equipment available at the nanofabrication lab (Center for Nano and Micro Manufacturing (CNM2)) at the University of California, Davis. The equipment used in this process included a wet bench for photoresist development and substrate cleaning, a Sky 335R6 Heated Roller Laminator (Oregon Laminations Company, Portland, OR, USA) for laminating the SUEX dry film, an EVG 620 Mask Aligner (Irvine, CA, USA) for exposing the photomask patterns, and a Dektak XT (Bruker Corporation, Billerica, MA, USA) for analyzing the step height of the photoresist and Ga_2_O_3_. Additionally, a PlasmaTherm Apex SLR RIE/ICP (Bruker Corporation, Billerica, MA, USA) was used for etching Ga_2_O_3_, a Carl Zeiss Axiotron Microscope (Zeiss Global Group Inc., Dublin, CA, USA) for optical inspection, an FEI Nova NanoSEM 430 (Bridge Tronic Global, Irvine, CA, USA) for microscopy analysis, a hotplate for heated resist stripping, and a programmable vacuum oven for post-exposure baking.

**Figure 1:**
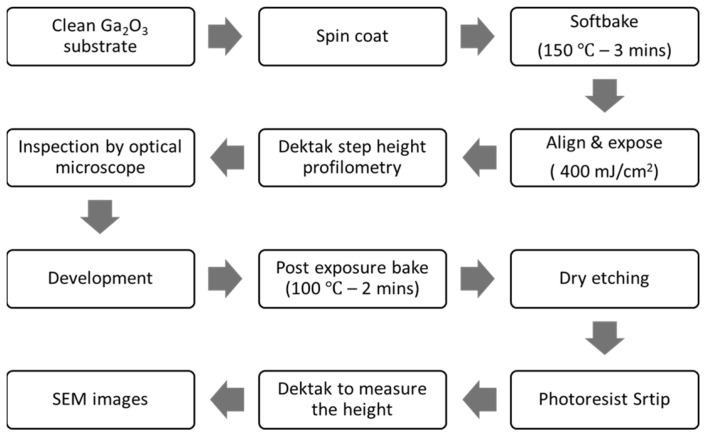
A flowchart illustrating the fabrication process of the etched Ga_2_O_3_ substrate.

Figure 1 presents a flowchart of the fabrication process of the etched Ga_2_O_3_ substrate. The etching process for Ga_2_O_3_ substrates necessitates thorough substrate cleaning. Initially, substrates of Sn-doped Ga_2_O_3_ (010) and ($\bar{2}01$) were prepared. A sequential ultrasonication process was employed to clean the substrates, utilizing acetone for 5 min, isopropanol for 5 min, deionized (DI) water for rinsing, and drying with nitrogen. After cleaning, we employed a negative photoresist, Futurrex NR9–1500PY (Futurrex, Inc., Franklin, NJ, USA), which was applied to the substrates via spin coating, and a SUEX K40 dry film (DJ MicroLaminates, Inc., Sudbury, MA, USA) that was laminated directly onto the surface.

The negative photoresist Futurrex NR9–1500PY was applied to the substrates through spin coating at a speed of 3000 rpm for 45 s. Given the requirement for a thicker resist in the etching process, the thickness of the photoresist was adjusted by varying the spin coater speed from 1500 rpm to 300 rpm, over durations ranging from 2 to 45 s. This adjustment also enabled control over the density and alignment of the Ga_2_O_3_ substrate.

**Figure 2:**
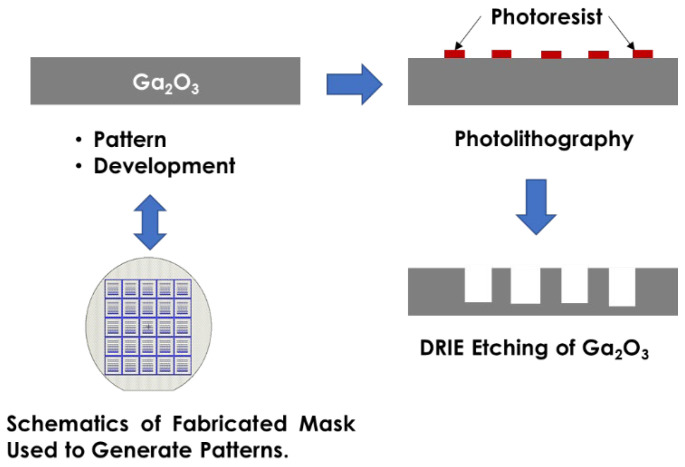
Schematic of the dry-etching process and fabrication of Ga_2_O_3_.

Subsequently, a soft bake was conducted at 150 °C for 2 min to enhance photoresist adhesion. The substrates were then aligned and exposed using the EVG 620 at an energy dose of 400 mJ/cm^2^ in a vacuum contact. A counter dummy Ga_2_O_3_ was used to achieve good planarization between the photomask and the sample. A post-exposure bake was performed at 100 °C for 2 min to further stabilize the photoresist patterns. The surface features were then examined using an optical microscope. Following this, deep reactive ion etching (DRIE) was employed to selectively remove undesired areas, thereby achieving the targeted shapes and openings in the Ga_2_O_3_ substrates (Figure 2).

We also employed an alternative approach by laminating SUEX K40 directly onto the Ga_2_O_3_ ($\bar{2}01$) substrates, with a thickness of 38.26 μm, as measured with Dektak. The substrates were then aligned and exposed using the EVG 620 at an energy dose of 700 mJ/cm^2^ in a vacuum contact. A counter dummy Ga_2_O_3_ was used to achieve good planarization between the photomask and the sample. A post-exposure bake was performed at 80 °C for 1 h and then cooled down overnight.

The Ga_2_O_3_ samples were etched using PlasmaTherm Apex SLR RIE/ICP. In the dry-etching process, BCl_3_ ran at 20 sccm for 5 min. The etching step included multiple loops with an inductively coupled plasma (ICP) power of 400 W and the power of RIE of 100 W. The cooling steps included 40 sccm Ar at 90 mTorr for 1 min. Finally, the photoresist was stripped overnight with a Futurrex RR41 resist stripper at 110 °C and 120 °C for NR9–1500PY and SUEX dry film, respectively. Finally, the samples were analyzed by using DektakXT and field-emission scanning electron microscopy (FEI 430 Nova NanoSEM).

### 2.2. Structural and Surface Morphology Characterizations

DektakXT was used to measure the depth profile and to prove the precise thickness of thin films across the wafer surface. In addition, the substrates were observed using optical microscopy and scanning electron microscopy, which provided images of the sample topography by scanning the surface with a high-energy beam of electrons.

## 3. Results

### 3.1. Surface Morphology

The surface features were first examined using an optical microscope, confirming the introduction of patterns. The characterization of optical images in surface lithography also requires high-resolution imaging techniques to ascertain the fidelity and precision of pattern transfer on Ga_2_O_3_ substrates.

**Figure 3:**
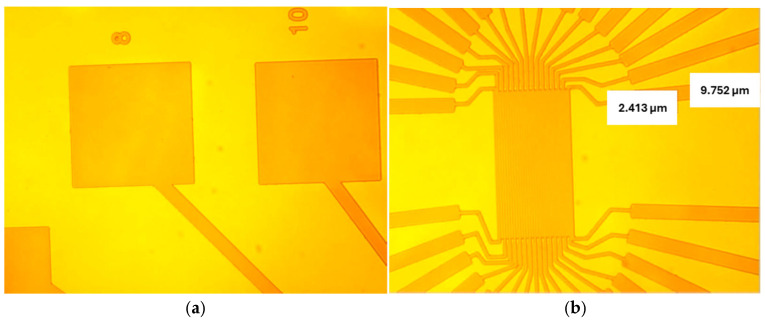
(**a**,**b**) Optical microscope images of Ga_2_O_3_ substrate lithography at different locations with NR9–1500PY negative photoresist.

**Figure 4:**
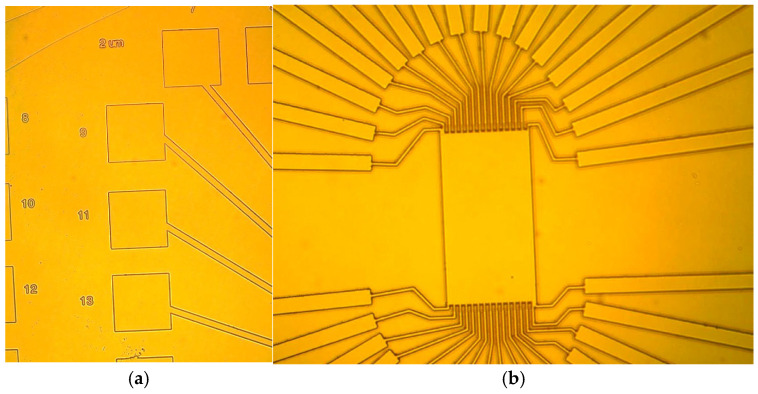
(**a**,**b**) Optical microscope images of Ga_2_O_3_ post NR9–1500PY negative photoresist stripping (1.65 μm etched Ga_2_O_3_ ($\bar{2}01$).

Figure 3 and Figure 4 show the optical response of the lithographically patterned surfaces, with clear differentiation of intended patterns of 2.41 μm and 9.75 μm (Figure 3b), confirming the successful introduction of specific geometric configurations.

We initially used NR78–8000P (Futurrex, Inc., Franklin, NJ, USA) as a photoresist, which led to poor patterning because of its thickness and ineffective wafer edge cooling. Despite BCl_3_’s good selectivity, the photoresist burned during a 30-min etch, and a hardbake step worsened the removal issues of the photoresist. For improved lithography, a counter piece was needed to ensure proper planarization of the photomask to the Ga_2_O_3_ piece, preventing uneven UV exposure. To address the resist burning, the hardbake step was removed, a thinner resist (NR9–1500P at 1.7 µm) was used, and the etch was segmented into 5-min increments with Ar cooling in between.

### 3.2. Etching Parameters

Etching parameters in lithography are crucial for achieving the desired results, particularly when creating deep-etched features. Factors that collectively determine the efficiency, rate, and quality of the dry-etching process for β-Ga_2_O_3_ include gas composition, ICP power, bias power, cooling water temperature, chamber pressure, and mask material.

We analyzed the effects of the crystal structure and the etching time and rate on the etched features of Ga_2_O_3_ using our etching process to improve the pattern transfer and minimize the undesired effects, such as undercutting or roughness.

#### 3.2.1. Crystal Orientation

Crystal orientation significantly influences etching outcomes in photolithography due to variations in atomic arrangements and bonding strength within the crystal lattice. Different crystallographic planes exhibit distinct etch rates, leading to anisotropic etching, where certain directions are preferentially etched over others. This anisotropy affects the fidelity of the final pattern, potentially causing issues such as unintended sidewall angles or undercuts. Therefore, understanding and controlling the crystal orientation of Ga_2_O_3_ is crucial for optimizing etching processes and achieving precise lithographic results.

**Figure 5:**
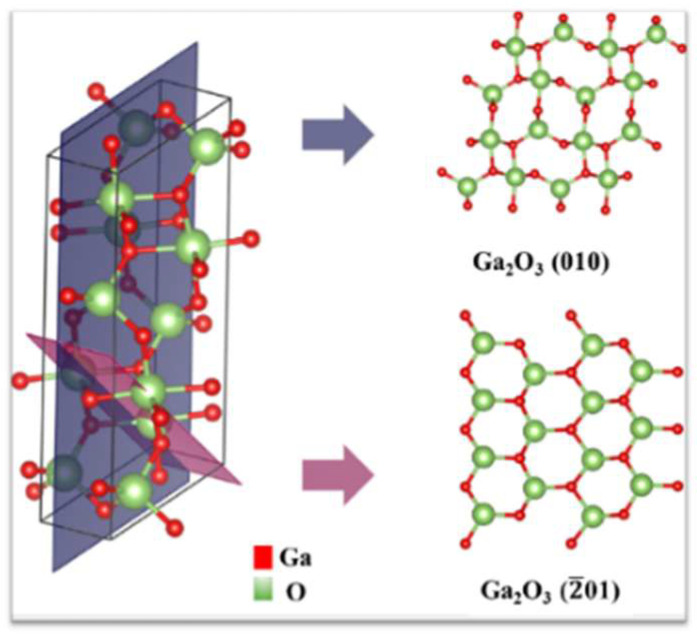
Crystal structure of β-Ga_2_O_3_ and 2D structures obtained after cleaving along (010) and ($\bar{2}01$) direction, as shown by blue and purple planes, respectively [26].

Anam et al. [26] analyzed the structural, thermal, and electronic properties of two-dimensional gallium oxide (β-Ga_2_O_3_) using a first-principles design. The atomic arrangement in the (010) and ($\bar{2}01$) surfaces of Ga_2_O_3_ is different (Figure 5), which affects the etching profile. The (010) plane has four Ga_2_O_3_ in its unit cell, with Ga atoms having only a tetrahedral coordination geometry. The atomically thin-layered structure in the ($\bar{2}01$) direction has slightly different structural parameters from the rest of the predicted 2D allotropes of β-Ga_2_O_3_. It has a 1 to 1 Ga and O ratio, with six Ga atoms and six O atoms in its unit cell. Therefore, the fidelity of the etched pattern is affected by the stability of the surface structure.

**Figure 6:**
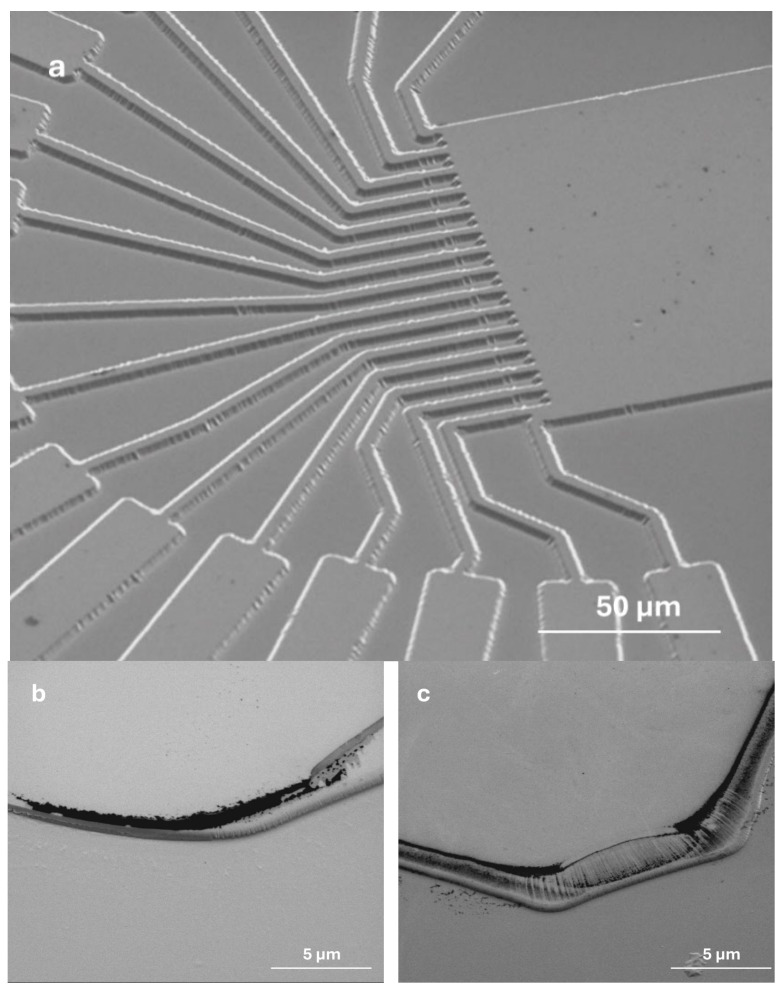
SEM images of ~200 nm etched Ga_2_O_3_ substrates post preliminary etch in 30 min etch: (**a**,**b**) ($\bar{2}01$); (**c**) (010).

The atomic arrangement in different directions on the Ga_2_O_3_ (010) and Ga_2_O_3_ ($\bar{2}01$) surfaces is shown in the morphology of the Ga_2_O_3_ substrate after dry etching, as presented in Figure 6 The SEM image of the Ga_2_O_3_ substrate at a low magnification (Figure 6a) shows the etched pattern that is clearly visible and recognizable. At a higher magnification (Figure 6b,c), the SEM images were distinctly different in the edge and corner profiles, although both the (010) and ($\bar{2}01$) orientations were etched at 6.7 nm/min for 30 min. The surface structure of Ga_2_O_3_ (010) is more stable than Ga_2_O_3_ ($\bar{2}01$) [26]. This difference in the surface energy and stability is clearly seen in Figure 6b,c, where the (010)-oriented Ga_2_O_3_ has well-defined corners compared with Ga_2_O_3_ ($\bar{2}01$).

Hogan et al. [21] investigated the dry etching of β-Ga_2_O_3_ and found that the etch rate on (100)-oriented Ga_2_O_3_ was significantly lower than on the (010) and ($\bar{2}01$) planes. This difference was attributed to the presence of surface oxygen anions and a lower concentration of dangling bonds on the (100) surface.

For wet etching, they used H_3_PO_4_, which is considered an efficient wet etchant on (100)-oriented β-Ga_2_O_3_ [11]. However, the etching process led to isotropic side etching. Despite the slower etch rate on the (010)-oriented Ga_2_O_3_, it may be the better choice for achieving high-aspect-ratio micro- or nanostructures. Notably, groove-shaped pits were observed on etched β-Ga_2_O_3_ (010) single crystals [12]. The (010) orientation of Ga_2_O_3_ tends to promote the formation of deeper micro- or nano-features on the surface, with the etch pit formation influenced by voids and dislocation defects.

In the context of two-dimensional (2D) β-Ga_2_O_3_ allotropes, the energy characteristics of the (010) and ($\bar{2}01$) planes reveal distinct differences in stability and electronic properties, which may influence their suitability in various semiconductor technologies. Potential future integration includes UV-responsive nanosensors utilizing a 4.8 eV bandgap for advanced photoenergy applications. This study examined the energetic stability of these planes and identified the structural configurations responsible for their unique electronic behaviors using particle swarm optimization combined with density functional theory (DFT) calculations [26]. The (010) plane exhibited a more favorable energetic profile compared with the ($\bar{2}01$) orientation, primarily due to strong bonding interactions and lower surface energy. This atomic arrangement in the (010) plane promotes optimal π-bonding, which enhances structural integrity and facilitates better electronic transport properties. In contrast, while the ($\bar{2}01$) plane remains stable, it has a higher energy configuration that could lead to increased electronic localization and reduced carrier mobility, potentially affecting device performance.

Band structure calculations, using both generalized gradient approximation (GGA) and the local density approximation (LDA–1/2), show that the (010) plane maintained a larger indirect band gap of approximately 4 eV [27]. This property is particularly advantageous for applications in transparent conductive oxides and semiconductor devices, as it supports improved light absorption and minimizes recombination losses.

In conclusion, the comparative analysis of the (010) and ($\bar{2}01$) planes of β-Ga_2_O_3_ highlights the importance of structural orientation in determining both the fidelity of the etch patterns and the electronic and optical characteristics of 2D materials.

#### 3.2.2. Etching Time

Etching time is another crucial parameter studied for ensuring accurate and reliable photolithographic outcomes. It is well known that if the etching process is insufficient, it can lead to incomplete pattern transfer, resulting in unwanted material remaining on the substrate. This not only compromises the patterns’ integrity but can also diminish the features’ resolution. Meanwhile, an excessive etching time can lead to over-etching, which may cause a loss of critical dimensions, distort the intended feature profiles, and introduce surface roughness, ultimately degrading the material quality. Adding BCl_3_ to the Cl_2_/BCl_3_ gas mixture can improve the etching rate of β-Ga_2_O_3_ to reduce the surface roughness after the etching reaction of BCl_3_/β-Ga_2_O_3_. As a result, the surface damage of β-Ga_2_O_3_ has been decreased after post-wet chemical treatments [22]. In the deep-etching process between BCl_3_ plasma and Ga_2_O_3_, the reaction of the etching mechanism leads to the dissociation of chloride radicals (Cl^−^) and the formation of volatile Ga-Cl species, which are absorbed and remove the undesired material. The oxygen molecules in Ga_2_O_3_ are removed and replaced by chloride (Cl) to react with Ga molecules. However, B atoms could form a non-volatile compound that stays on the etched surface as a residue. Therefore, it would be impossible for boron to be an effective incorporation in increasing the etching mechanism.

**Figure 7:**
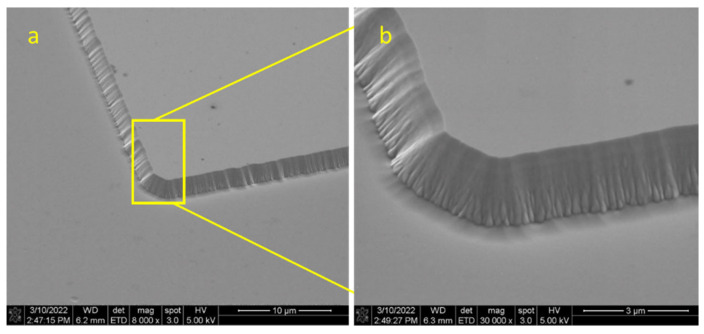
Optical microscope images of Ga_2_O_3_ substrate lithography at different locations with NR9–1500PY negative photoresist. (**a**) Top view of definedsquared pads and (**b**) schematic of micro-array contacts.

**Figure 8:**
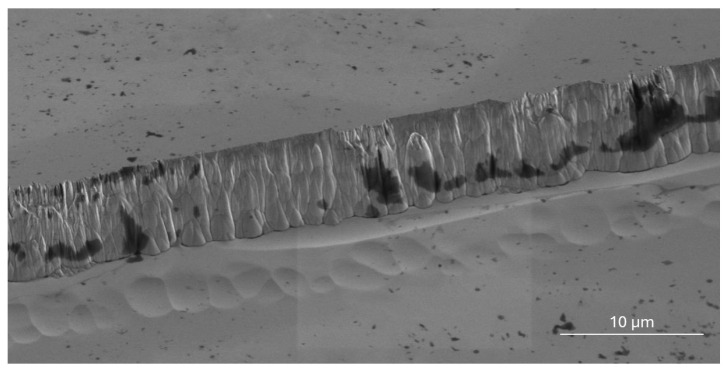
Sidewall of 6.96 µm etched ($\bar{2}01$) Ga_2_O_3_ after 24 h.

Figure 7 and Figure 8 show a top-view SEM image of the etched sidewall on Ga_2_O_3_ ($\bar{2}01$), with 1.65 µm after 5 h of etching (Figure 7), and 6.97 µm after 24 h of etching (Figure 8). Depths were averaged from profilometer scans at five locations per sample to confirm uniformity, with the maximum recorded value of 6.97 µm. With further optimization of the lithography and etch recipes, the surface can be cleaned, the sidewall angle can be made steeper, and the surface roughness can be reduced.

Following the etching process and subsequent treatment with Futurrex RR41 (110–120 °C overnight), we evaluated piranha cleaning (H_2_SO_4_–H_2_O_2_; 5 min) and O_2_ plasma ashing (200 W; 2 min) protocols. The plasma-ashing process ensures the removal of the photoresist from the etched substrates. Consequently, this process reduced surface residue by approximately 70%; however, complete removal was not achieved, and harsher conditions pose a risk of surface degradation. For harsh sensors, this residue may enhance passivation.

#### 3.2.3. Etching Rate

The etching rate in DRIE directly impacts the depth, aspect ratio, and quality of the etched features. A balanced etching rate is essential for deep etching: too fast a rate can lead to isotropic etching, rough surfaces, and a poor sidewall profile; too slow a rate can lead to long process times, surface non-uniformity, and reduced throughput. When comparing pure BCl_3_ and Cl_2_ etching, pure BCl_3_ was much more effective than pure Cl_2_ [21]. The etch rate using mixtures of BCl_3_ and Cl_2_ was roughly related to the weighted average of each etchant, indicating minimal interaction between the etch species. Specifically, BCl_3_ produced higher etch rates compared with Cl_2_, and the addition of Cl_2_ to BCl_3_ did not significantly enhance the etch rate [21,22].

The 24-h total process time included 211 cycles of 5-min BCl_3_ etching (1055 min; ~17.6 h) with 1-minute Ar cooling steps per cycle (~3.5 h), resulting in an etch depth of 6.96 μm for ($\bar{2}01$) Ga_2_O_3_. The etch rate of 6.7 nm/min represents the rate during active BCl_3_ etching periods, while the reported process durations (e.g., 5 h and 24 h) incorporate the full cyclic procedure, including 1-min Ar cooling steps per cycle, leading to a slightly lower effective average rate of ~6.6 nm/min in extended runs. The etch rate for SUEX K40 during the same 5 min cycle was 2.44 nm/min (12.2 nm/cycle for 211 cycles). The Ga_2_O_3_-to-SUEX K40 selectivity was 2.75:1. Table 1 shows different etching rates obtained in the literature compared with our work.

**Figure 9:**
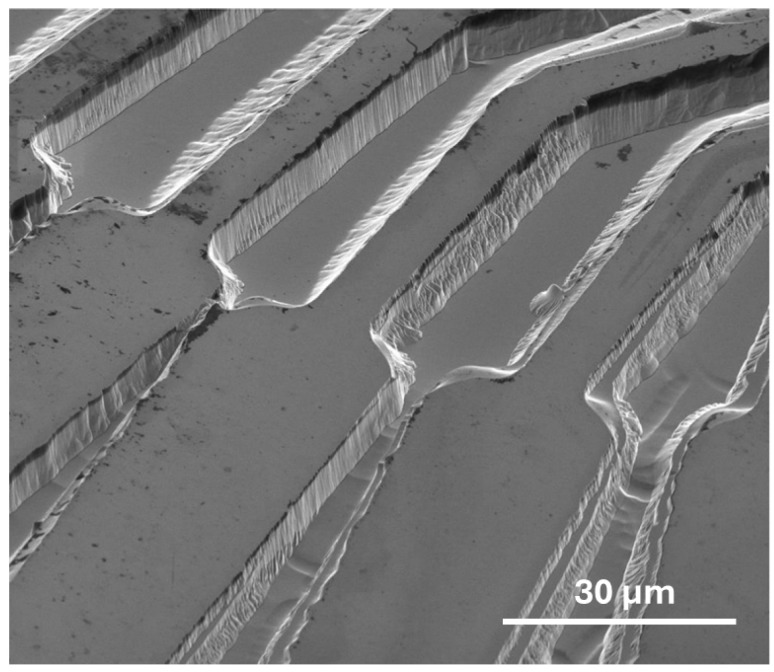
SEM image of 6.97 μm etched ‘interconnect’ features on Ga_2_O_3_ ($\bar{2}01$).

A consistent etching rate is crucial for achieving uniform feature sizes and depths across the entire wafer. However, the pattern angle can affect the steepness of the sidewalls in certain regions. As shown in Figure 9, surface features positioned at different angles exhibit varying degrees of sidewall roughness, which may also be linked to the underlying crystal orientation. Additionally, some areas show a ‘stepped’ appearance, as though multiple lithographic steps were performed, though this is not the case. The sidewall quality, including roughness and steepness, varies across features placed at specific angles, which may be influenced by crystal orientation. This suggests that crystalline orientation plays a role in determining the steepness and roughness of the sidewalls, even when the etching rate remains constant.

## 4. Conclusions

This study introduced an innovative deep ion-etching process for β-Ga_2_O_3_, achieving a remarkable etch depth of 6.97 µm on Ga_2_O_3_ ($\bar{2}01$) substrates, a significant advancement over previously reported etching techniques. The novelty of this work lies in the successful implementation of a BCl_3_-based inductively coupled plasma (ICP) reactive-ion etching (RIE) process, optimized with a SUEX K40 dry film photoresist to overcome lithographic challenges associated with small substrates (<5.00 mm × 5.00 mm). This approach eliminates edge bead issues, ensuring intimate photomask contact and enabling high-resolution patterning of features as small as <5.00 µm. The achieved etch rate of 6.70 nm/min, coupled with a Ga_2_O_3_ to SUEX K40 selectivity of 2.75:1, represents an advanced step in fabricating high-aspect-ratio nanostructures for vertical Ga_2_O_3_ devices.

Furthermore, this research provides novel insights into the influence of crystallographic orientation on sidewall quality, revealing that the (010) plane’s stability enhances pattern fidelity compared with the ($\bar{2}01$) plane.

While further optimization of etch parameters is needed to enhance sidewall verticality and smoothness, these results mark a significant leap forward in Ga_2_O_3_ nanofabrication. By enabling deep etching for applications in optoelectronic devices and harsh-environment nanosensors, this work not only expands the understanding of β-Ga_2_O_3_’s potential in 2D material systems but also sets a new benchmark for precision and scalability in wide-bandgap semiconductor processing, opening avenues for innovative electronic and energy technologies.

This research was motivated by the need for efficient fabrication processes, particularly for applications requiring sensors to operate in harsh environments, owing to their low leakage current density in power devices.

## Figures and Tables

**Table 1:** A comparison of the measured (this work) and reported results of Ga_2_O_3_ after dry etching.

Plasma Components	Ga_2_O_3_ Orientation	Etch Rate (Å/min)	Comments	Ref.
This work				
BCl_3_	(010) ($\bar{2}01$)	67		
Other works				
Cl_2_, BCl_3_, Cl_2_/BCl_3_	(010) ($\bar{2}01$)	120	Etch rate obtained for BCl_3_. Faster rates for (010) and ($\bar{2}01$) than (100).	[21]
Cl_2_/BCl_3_	(001)	-	The addition of BCl_3_ to Cl_2_ increases the etch rates of β-Ga_2_O_3_ and reduces the surface damage to produce a suitable performance of device.	[22]
BCl_3_/Ar	($\bar{2}01$)	700	Annealing found to repair the barrier height after etching.	[28]
Cl_2_/Ar, BCl_3_Ar	($\bar{2}01$)	120–700	To reduce damage in Schottky diodes.	[29]
BCl_3_/Ar	(001)	-	Surface quality improvement mechanism of ICP etching for Ga_2_O_3_ Schottky barrier diode.	[30]
BCl_3_/Ar	(100)	-	To investigate dry etching of the NiO/Ga_2_O_3_ heterojunction system.	[31]

## Data Availability

The raw data supporting the conclusions of this article will be made available by the authors on request.
